# Occult periprosthetic femoral fractures occur frequently during a long, trapezoidal, double-tapered cementless femoral stem fixation in primary THA

**DOI:** 10.1371/journal.pone.0221731

**Published:** 2019-09-19

**Authors:** Ho Hyun Yun, Jung Taek Lim, Se-Hyun Yang, Phil Sun Park

**Affiliations:** Department of Orthopaedic Surgery, Seoul Veterans Hospital, Seoul, South Korea; University of Ulsan College of Medicine, Asan Medical Center, REPUBLIC OF KOREA

## Abstract

The present study aimed to investigate the prevalence and clinical consequences of occult intra-operative periprosthetic femoral fractures in total hip arthroplasty (THA). Between 2012 and 2017, a total of 113 primary THAs were enrolled. The mean age of the patients was 66.4 ± 7.6 years. We assessed occult intra-operative periprosthetic femoral fractures with the use of computed tomography (CT) and risk factors, including age, sex, body mass index, diagnosis, stem size, and radiographic parameters of proximal femoral geometry were analyzed. We also assessed the differences in thigh pain and stem subsidence and alignment between the patients with and without occult periprosthetic femoral fracture. Occult intra-operative periprosthetic femoral fractures were found in 13 of 113 hips (11.5%). In 9/13 (69.2%) of occult fractures, fracture lines were started from the region below the tip of the lesser trochanter. Six periprosthetic femoral fractures (5.3%) were found during the operation. Out of the five hips that had detected femoral fractures around the lesser trochanter intra-operatively, four hips (80%) showed concurrent occult fractures on different levels. The female sex *(P =* .*01)* and canal filling ratio at 7 cm below the tip of the lesser trochanter *(P =* .*01)* were significantly different between the patients with and without occult periprosthetic femoral fracture. The sex was significantly associated with an increased risk in predicting an occult intra-operative periprosthetic femoral fracture (odds ratio of male, 0.25 compared with the female; 95% CI, 0.08–0.85; *p =* .*02*). There was a significant difference in the incidence of thigh pain between occult fracture group and non-occult fracture group *(P <* .*05)*. There were no significant differences in stem subsidence and alignment between the patients with and without occult periprosthetic femoral fracture. All 13 cases of occult intra-operative periprosthetic femoral fractures were healed at the final follow-up. Occult periprosthetic femoral fractures are common during a long, trapezoidal, double-tapered cementless femoral stem fixation in primary THA, that CT scans are helpful to identify them, and that these fractures do not adversely affect the implant’s survival if a rigid fixation of the implants has been achieved.

## Introduction

The number of periprosthetic femoral fractures during total hip arthroplasty (THA) is becoming much higher as a result of widespread use of cementless femoral stems. [1] Press-fit impaction has been the most popular technique for the fixation of cementless femoral stems, which may lead to periprosthetic femoral fractures. [1, 2]

The incidence of periprosthetic femoral fracture with cementless femoral stems during primary THA has been reported to be 3.5–5.4%, [3–6] whereas the rate of periprosthetic acetabular fracture with cementless acetabular cups is less than 1%. [7–9] Several studies [9–11] introduced occult intra-operative periprosthetic acetabular fractures, defined as those that were unrecognized during surgery, undetectable on the postoperative radiographs, and only diagnosed on the postoperative computed tomography (CT) images, as an unknown side effect of the press-fit techniques in primary THA, whereas occult intra-operative periprosthetic femoral fractures have received little attention in the literature.

We are not aware of any previous studies describing occult intra-operative periprosthetic femoral fractures. In an effort to improve our understanding of this issue, we assessed occult intra-operative periprosthetic femoral fractures during primary THA with the use of CT. [12] The purpose of this study was to investigate the prevalence of occult intra-operative periprosthetic femoral fractures and to determine the risk factors associated with them. We also evaluated the effect of occult intra-operative periprosthetic femoral fractures on implant survival.

## Materials and methods

**Fig 1:**
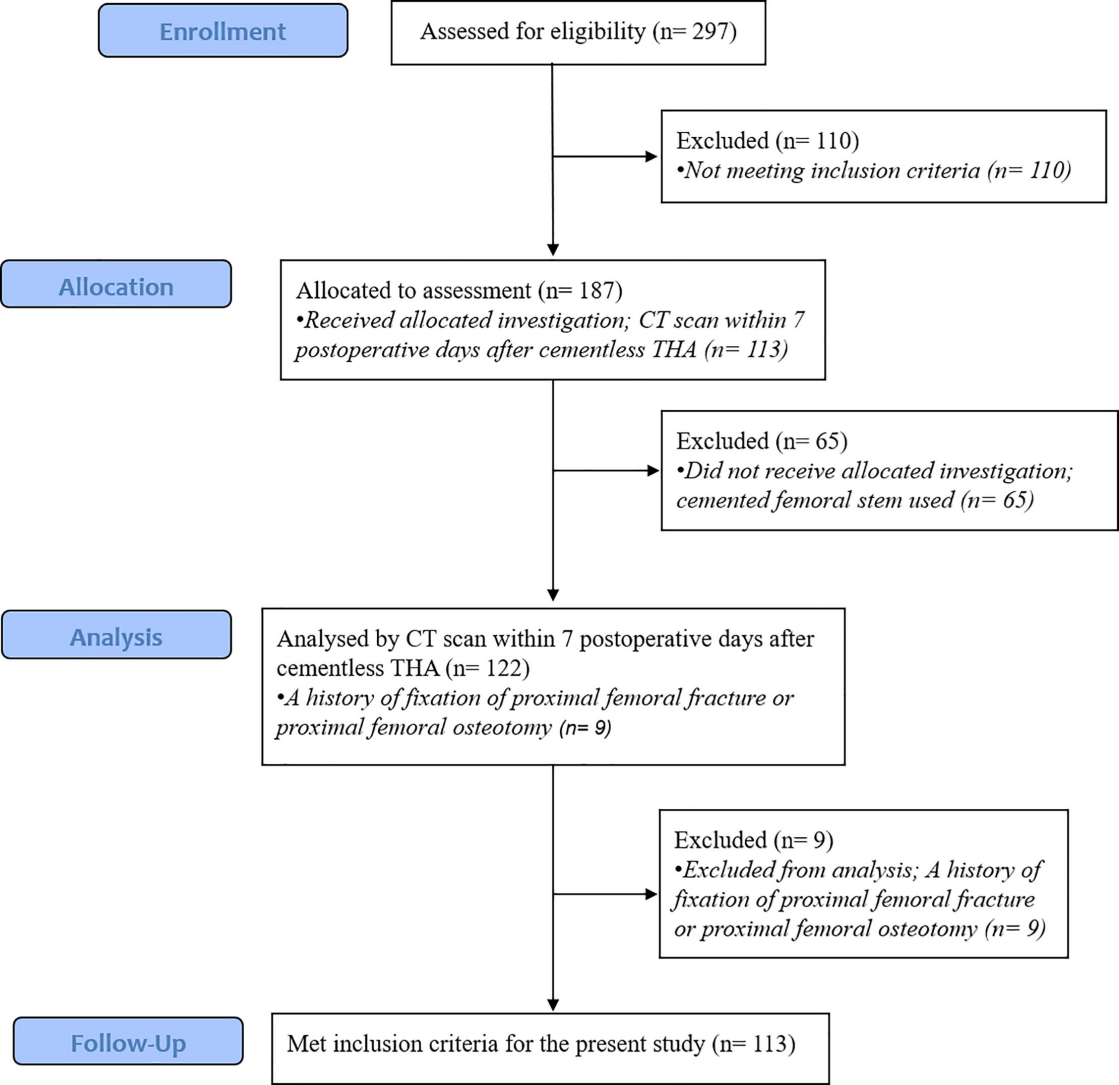
CONSORT guidelines patient flow diagram.

This study was retrospective. Patients who underwent primary THA between March 2012 and October 2017 (246 patients/297 hips) with a minimum of 12 months follow-up were the subjects of the present study. Other study protocols involved the routine examination of postoperative CT images for the purpose of determining the orientation of the cups and stems in primary THA. Accordingly, we obtained post-operative CT images in patients with primary THA. The use of the CT images in this study was approved by our institutional ethical committee (veterans health service medical center, study No. 2018-11-003). Patients (158 patients/187 hips) who were operated with the use of cementless femoral stems, underwent postoperative CT scans taken within one week after the operation and had preoperative and postoperative anteroposterior (AP) and cross-table trans-lateral (CTL) hip radiographs were taken enrolled in this study. Exclusion criteria for patients (58 patients/74 hips) in this study included a cemented femoral stem used and a history of fixation of proximal femoral fracture or proximal femoral osteotomy. We finally enrolled 113 hips (100 patients; 81 males and 19 females) in the present study. Patient flow-chart is shown as Fig 1. The mean age of the patients was 66.4 ± 7.6 years (range, 39–87), and the mean BMI was 24.5 ± 3.4 kg/m^2^ (range, 13.5–36.9). Preoperative diagnoses included osteonecrosis of the femoral head in 77 hips, dysplastic hip in 13 hips, osteoarthritis in nine hips, posttraumatic osteoarthritis in five hips, femur neck fracture in five hips, and other diagnoses in four hips. The mean follow-up was 37.2 months (range, 12–78 months).

The Corail^®^ stem (DePuy Synthes, West Chester, PA, USA) [13–16] was used as the femoral component implanted in all 113 hips. The Corail^®^ stem is made of grit-blasted titanium alloy (TiAl6V4). The primary mechanical stability of the Corail^®^ stem is achieved by (1) being double-tapered in the sagittal and coronal plane with a trapezoidal cross-section in the proximal part, which induces a wedge effect to give rotational stability and self-lock, (2) press-fit by 0.3 mm (0.15 mm per side) thickness of hydroxyapatite (HA) coating on the entire external surface, and (3) a quadrangular cross section in the distal part, which enhances rotational stability.

All operations were undertaken through a posterolateral approach by one surgeon (YHH). After cup fixation, the femoral canal was prepared by compaction broaching technique. Broaching was done sequentially until longitudinal and rotational stability was achieved. We estimated the stem alignment using a c-arm to prevent malalignment. The neutral stem position was defined as within 3° of valgus or varus stem alignment. If the estimated stem alignment was not a neutral position, we performed adjustment procedures. The size of the true femoral stem corresponded to the size of the last femoral broaching used. We used stems of size nine in one case, 10 in five, 11 in 13, 12 in 31, 13 in 21, 14 in 14, 15 in 13, and 16 in 15 cases. We checked the stability of the stems after implantation. At that time, all femoral stems showed no detectable motion between implant and bone.

Preoperative and postoperative radiographs were taken using a previously described protocol [17]. Every patient was supine, and the femurs were held in 15 degrees of internal rotation during imaging. The x-ray beam was centered at the midpoint between the superior margin of the symphysis pubis and the midpoint between the anterior superior iliac spines in the AP hip radiographs. In the CTL hip radiographs, the x-ray beam was directed parallel to the table, oriented 45 degrees cephalad from inferomedial to superolateral, and centered at the femoral head. Patients followed-up immediately postoperatively, and then at 3 days, 2 weeks, 4 weeks, 3 months, 6 months, 1 year, and then yearly thereafter. At each follow-up evaluation, all patients were asked to complete a self-administered questionnaire. Dual-energy CT (DECT) scans were obtained with a dual-source CT system (Somatom Definition Flash; Siemens Healthcare, Forchheim, Germany). Tube voltages were set at 100 kVp and 140 kVp with an activated tin filter. DECT acquisition was performed using a detector configuration of 32 x 0.6 mm, pitch of 0.6, rotation time of 0.5 s, and effective milliampere second value of 160 mAs with automated attenuation-based tube current modulation. To decrease the artifacts of large metal part in postarthroplasty patients by detecting the hyperdense artifacts and compensating the image with designated algorithm, reconstruction were performed using a metal artifact reduction software (iMAR; Siemens Healthcare, Forchheim, Germany) with dedicated parameters optimised for large metallic implants. The iMAR algorithm was applied in post-processing. For the algorithm, the type of iMAR filter was selected for patients based on their implant type. All radiographic and CT image data were stored in a server using a Picture Archiving and Communication System (Infinitt, Seoul, South Korea).

**Fig 2:**
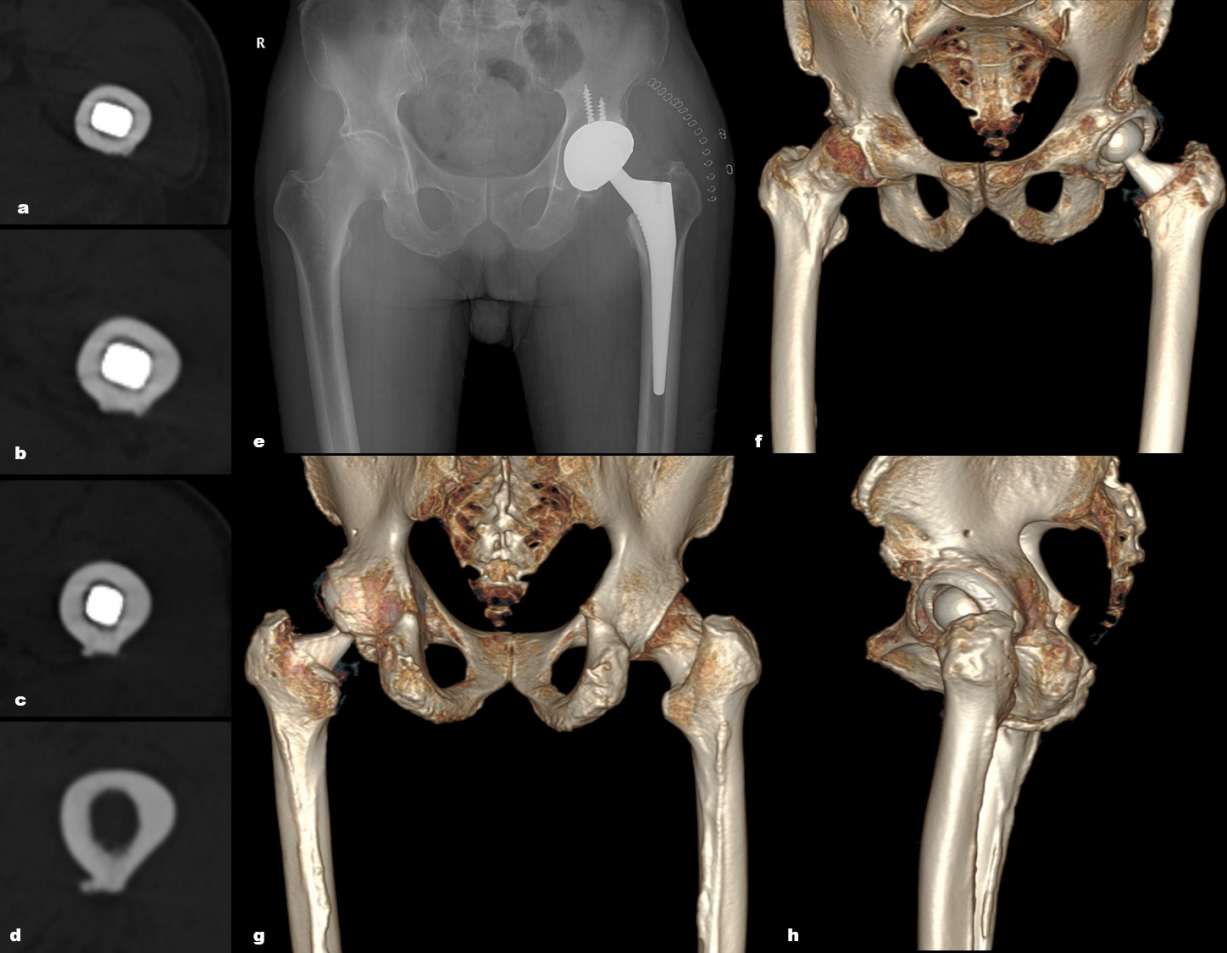
The diagram shows post-operative CT images (a-d and f-h) and a radiograph (e) for a 74-year-old man. (a-e) transverse radiolucent lines were seen on axial CT images along the corner of implant. (e) a post-operative and (f-h) 3-dimensonal reconstructed CT images (anterior, posterior, and lateral) show that there is no fracture lines around the implant.

**Fig 3:**
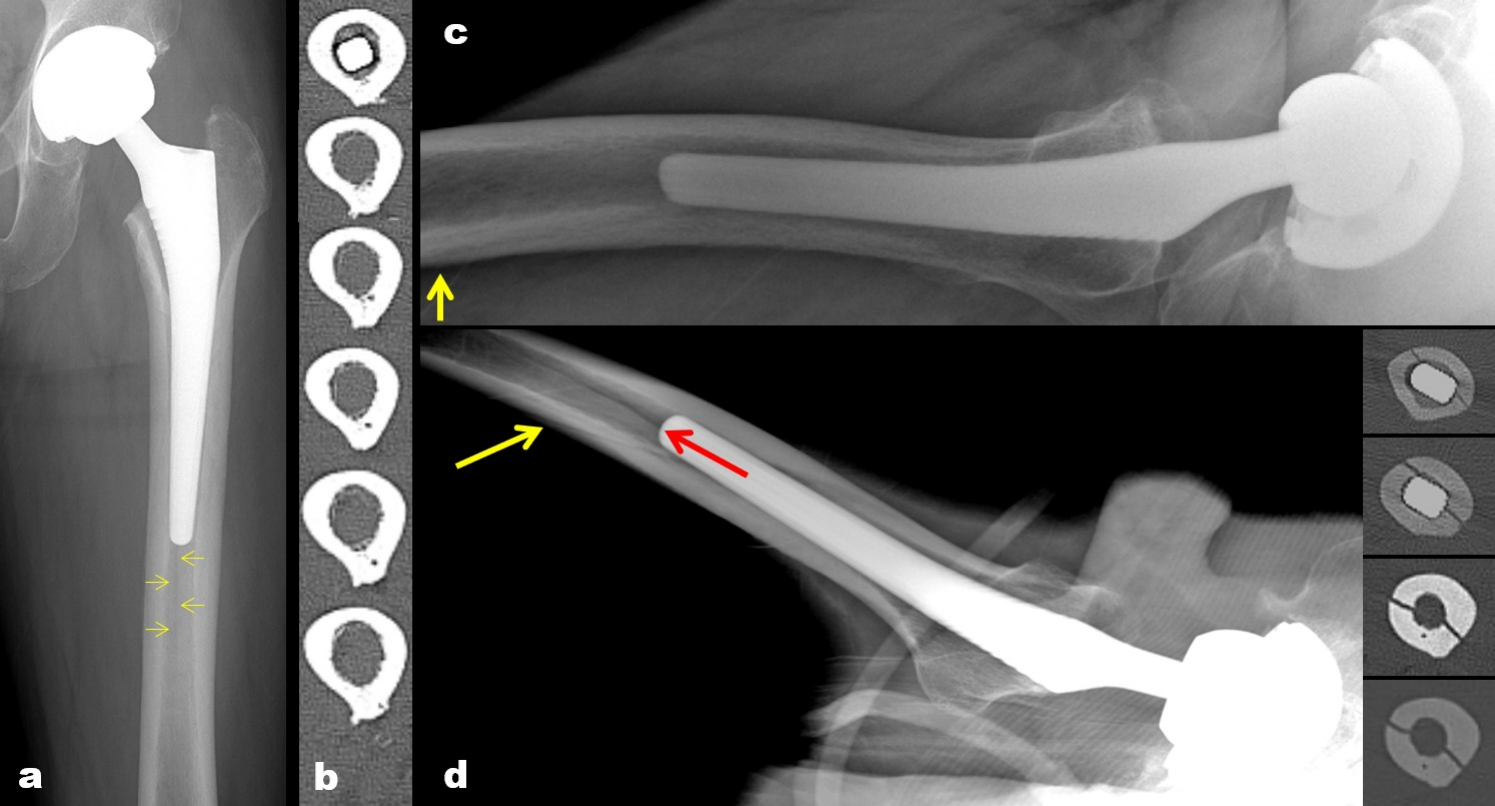
We defined nutrient artery canal of the femur (yellow arrow) as (a) a longitudinal radiolucent line traversing the medullary cavity or (c) an oblique radiolucent line seen traversing the cortex on radiographs. (b) Subsequent axial CT images from proximal to distal show a hypodense line having all of the following three properties in the posterior cortex was accepted as a nutrient canal on CT images: (1) an outer ostium on the outer cortical surface, (2) an uninterrupted course through the cortex, and (3) an inner ostium opening to the medullary cavity on the inner surface of the cortex. (d) We were able to confirm a fracture line (red arrow) in the axial CT images.

We observed periprosthetic femoral fractures on immediate postoperative radiographs and CT scans. A fracture was diagnosed when a fracture line was confirmed in AP or CTL hip radiographs or in the axial CT images. 3-dimensional reconstruction images were helpful to differentiate fractures from metal artifact (Fig 2). We distinguished fracture lines from nutrient artery canals of the femur on radiographs [18] and CT images [19] (Fig 3). One of the authors screened radiographs and CT scans of 113 hips, and the other authors reviewed the detected periprosthetic femoral fractures. All the authors were skillful at applying all tools of image adjustments.

We classified the intra-operative periprosthetic femoral fractures according to Capello et al. [4] We defined occult intra-operative periprosthetic femoral fractures as those that were unrecognized during surgery, undetectable on the postoperative radiographs, and only diagnosed on the post-operative CT images. Patients with intra-operative femoral fractures recognized during surgery were allowed non weight-bearing ambulation on the affected side for 3 weeks, and patients with occult intra-operative femoral fractures observed on the CT scans began progressive weight-bearing ambulation as tolerated on the day after surgery with no alteration of their routine postoperative protocol except for more frequent monitoring with serial radiographs of 1-week interval during the postoperative 4 weeks.

A diagnosis of thigh pain was made according to the definition by Barrack et al. [20] Thigh pain was considered present when a patient had a pain on the anterior and/or lateral thigh below the inguinal area. When the patient had a pain over the posterior thigh or gluteal region or pain that radiated to the lower leg, we reviewed lower lumbar and sacral spines on postoperative CT scan. When arthrosis of the spine was seen, spinal pathology was thought to be the etiology of pain. The intensity of thigh pain, if present, was measured on the visual analog scale (VAS).

Radiographic measurements for stem subsidence [21] and alignment [22] were performed using the postoperative 3 days and the final follow-up AP hip radiographs. Pre-operative AP hip radiographs were used to evaluate the proximal femoral geometry, including canal flare index (CFI) [23], canal-calcar ratio (CCR) [24], and canal bone ratio (CBR) [25]. Post-operative AP hip radiographs were used to assess the amount of canal filling of the stem, known as canal fill ratio (CFR) [26] at 2 points: 2 cm above and 7 cm below the tip of the lesser trochanter. These measurements were analyzed by a single observer, who was not involved in the treatment and unaware of the present study design. All measurements were performed digitally using the ruler function on the Picture Archiving and Communication System (PiViewStar Version 5080, Infinitt, Seoul, Korea).

Descriptive data were analyzed in term of the mean ± standard deviations (SD) for continuous variables and frequencies or percentages for categorical variables. Four hips (number of the hip in diagnosis was < 3) were excluded, and a total of 109 hips were included in the statistical analysis. Normality tests were performed with the Shapiro-Wilk test. Clinical (age, sex, body mass index [BMI], preoperative diagnosis), surgical (stem size), and radiographic (CFI, CCR, CBR, CFRs at 2 cm above and 7 cm below the tip of the lesser trochanter) parameters were analyzed to assess any risk factors for an occult intra-operative periprosthetic femoral fracture. Thigh pain and stem subsidence and alignment were analyzed to assess the differences between the patients with and without occult periprosthetic femoral fracture. Continuous variables were analyzed using the Mann Whitney U test because the assumptions of the data were not satisfied whereas categorical variables were analyzed using Fisher’s exact test. Stepwise logistic regression was used to determine the most important predictors of an occult intra-operative periprosthetic femoral fracture among variables. All statistical analyses were performed using SPSS software, versions 18.0 (SPSS Inc, Chicago, USA) and R 3.5.1 (R Development Core Team; R Foundation for Statistical Computing, Vienna, Austria). P values < 0.05 were considered to indicate statistical significance.

## Results

**Fig 4:**
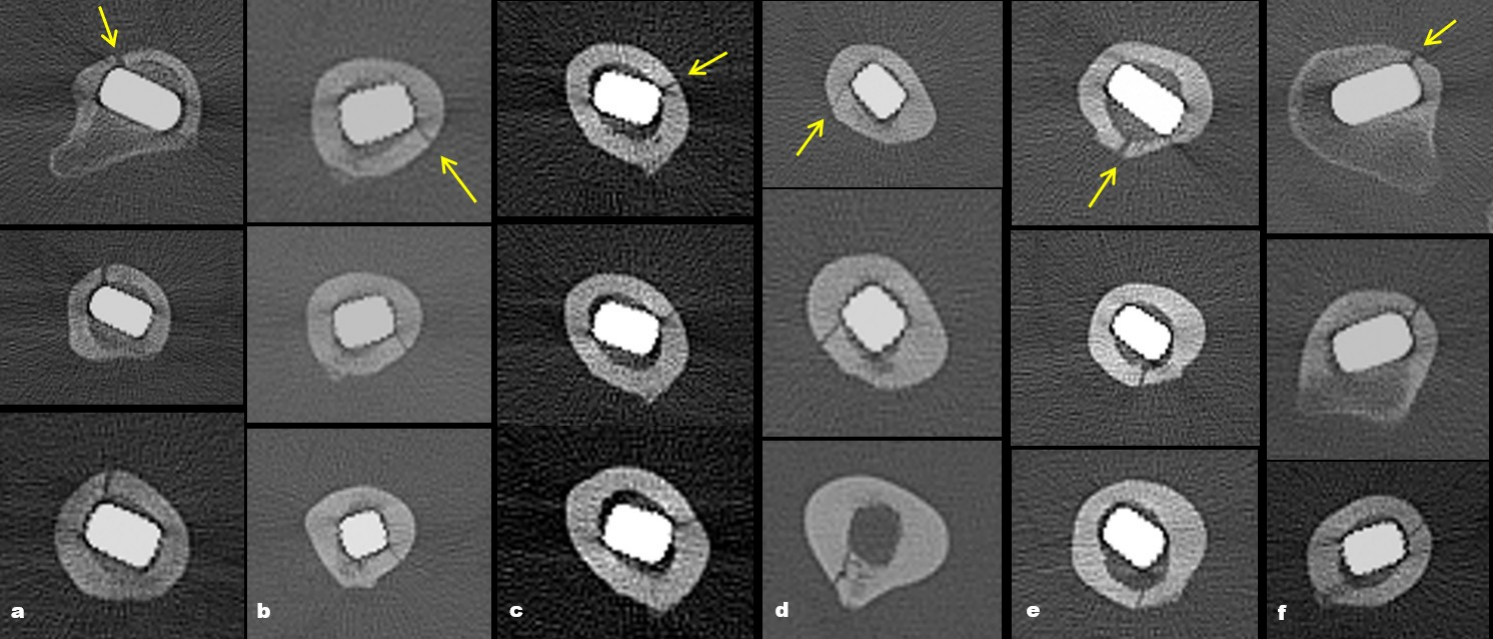
The diagram shows the various direction of obliquity and length of occult intra-operative periprosthetic femoral fractures on axial CT images. The arrow on the images indicates the location of the proximal entry point in each fracture line.

**Fig 5:**
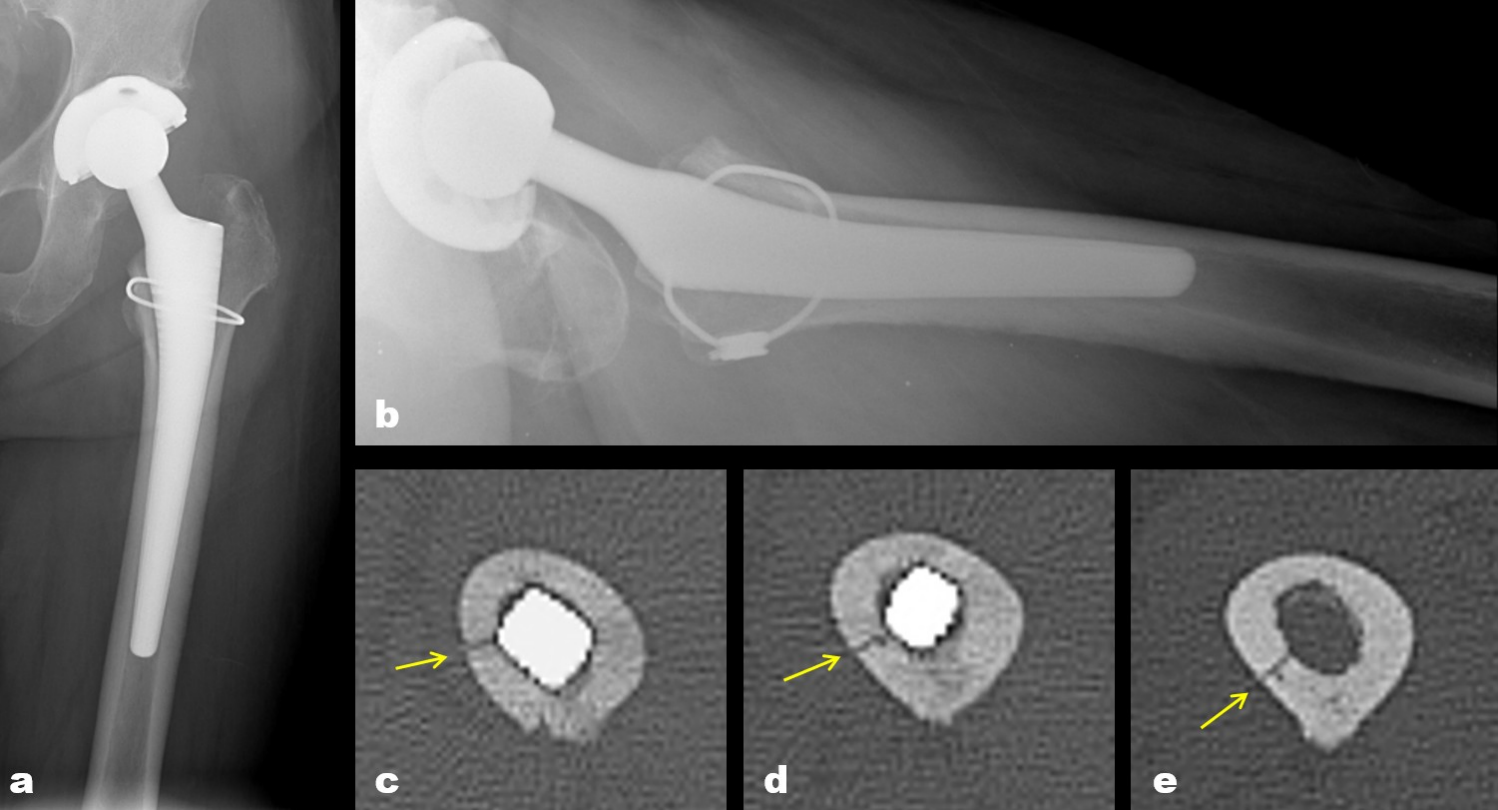
The diagram shows post-operative radiographs (a and b) and CT images (c-e) for a 72-year-old woman. (a and b) A type TL periprosthetic femoral fracture was detected during operation and fixed by a cerclage wiring. (c-e) At the same time, an occult intra-operative periprosthetic femoral fracture (arrow) was seen on axial CT images on a different level.

Occult intra-operative periprosthetic femoral fractures were found in 13 of 113 hips (11.5%). In 9/13 (69.2%) of occult fractures, fracture lines were started from the region below the tip of the lesser trochanter (Table 1). Visible patterns of occult intra-operative periprosthetic femoral fractures were shown in Table 1 and Fig 4. In addition, six periprosthetic femoral fractures (5.3%) were found during the operation. Of those, one was type T_G,_ four were type T_L_, and one was type A_1_ periprosthetic femoral fracture. There were four hips which showed concurrently both occult and non-occult periprosthetic femoral fractures on different levels (Fig 5). Of those hips, the types of non-occult periprosthetic femoral fractures were three type T_L_ and one type A_1_ periprosthetic femoral fracture. One type B_1_ periprosthetic femoral fracture (0.9%) was seen on the postoperative radiographs (Fig 3D).

**Table 1:** Visible patterns of occult intra-operative periprosthetic femoral fractures on axial CT images.

No.	Sex	Age	Direction (proximal/distal)	Length (caudal/cranial)
**1**	F	63	anterocentral/anterolateral	below the lesser trochanter/around the stem tip
**2**	F	65	anteromedial/anteromedial	above the lesser trochanter/middle thirds of the stem
**3**	M	67	posterocentral/aosterocentral	tip of the lesser trochanter/distal thirds of the stem
**4**	F	72	postermedial/posteromedial	subtrochanteric/around the stem tip
**5**	M	65	anteromedial/anteromedial	above the lesser trochanter/subtrochanteric
**6**	M	67	anteromedial/anteromedial	above the lesser trochanter/middle thirds of the stem
**7**	M	67	anteromedial/anteromedial	tip of the lesser trochanter/distal thirds of the stem
**8**	M	71	anterolateral/anterolateral	below the lesser trochanter/distal thirds of the stem
**9**	F	74	anteromedial/posteromedial	middle thirds of the stem/below the stem tip
**10**	F	78	anteromedial/anteromedial	below the lesser trochanter/distal thirds of the stem
**11**	M	39	anteromedial/anteromedial	above the lesser trochanter/subtrochanteric
**12**	F	63	anteromedial/anteromedial	middle thirds of the stem/distal third of the stem
**13**	F	72	anteromedial/posteromedial	middle third of the stem/below the stem tip

**Fig 6:**
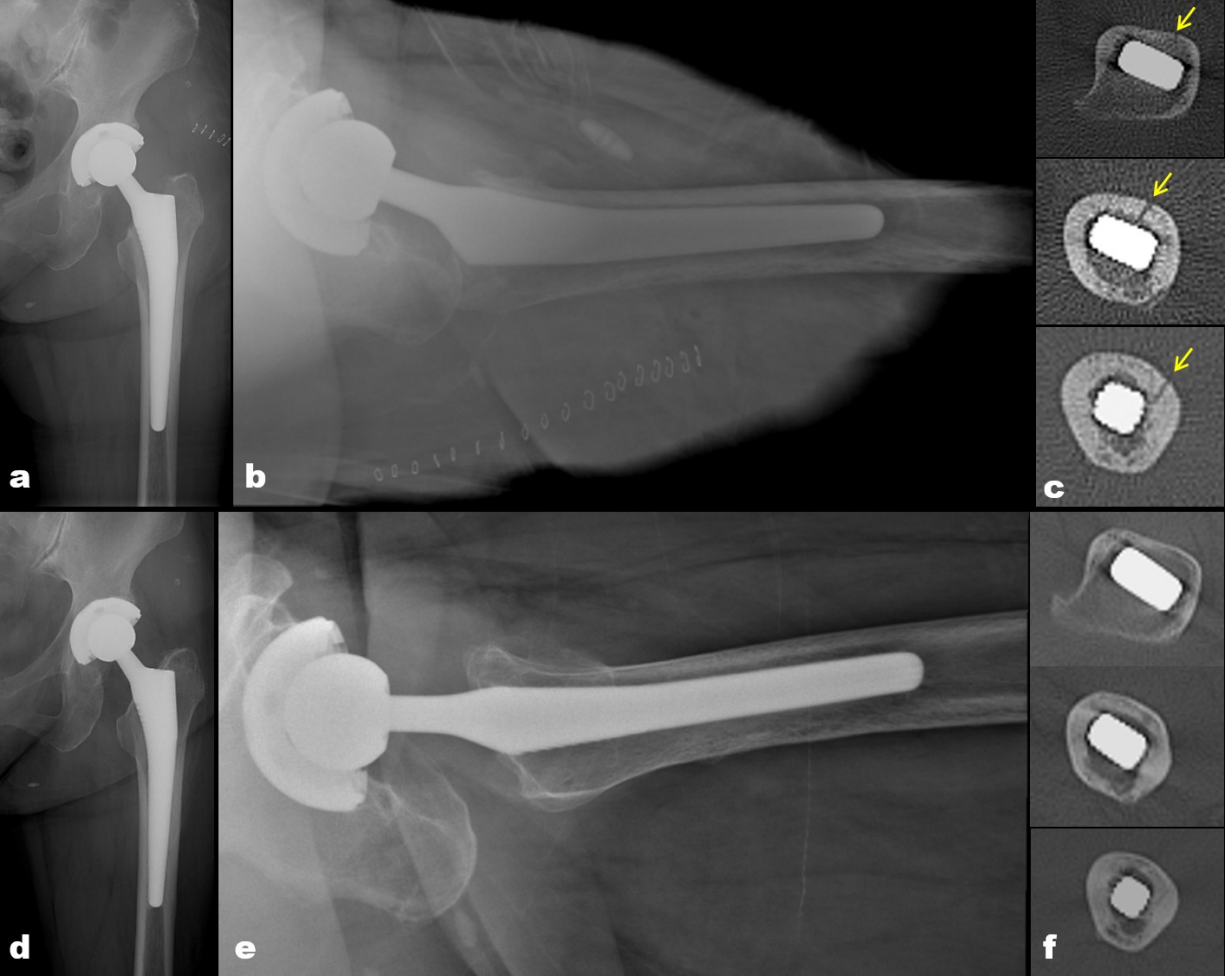
The diagram shows post-operative radiographs (a, b, d and e) and CT images (c and f) for a 63-year-old woman. An occult intra-operative periprosthetic femoral fracture (arrow) was seen on the three days post-operative (c) axial images. The patient had no additional treatment except for partial weight bearing ambulation on the affected side for 4 weeks. Achieved bone union was seen on the three years post-operative (f) axial images. Comparing radiographs between (a and b) initial post-operative and (d and e) three years post-operative radiograph, the implant had bone ingrowth with no malalignment.

17/113 patients (15.0%) (occult fracture group; 9/13 [69.2%], non-occult fracture group; 8/100 [8.0%]) reported thigh pain during the follow-up period. There was a significant difference in the occurrence of thigh pain between occult fracture group and non-occult fracture group *(P <* .*05)*. The median time of pain onset was postoperative 7 days in the occult fracture group and postoperative 3 months in the non-occult fracture group. In 14 patients (82.4%, 14/17), thigh pain was relieved during the follow-up. However, in the remaining 3 patients (occult fracture group; 1/13 [7.7%], non-occult fracture group; 2/100 [2.0%]) thigh pain persisted until the latest follow-up. There was no significant difference in the occurrence of persistent thigh pain between occult fracture group and non-occult fracture group. The maximum VAS score during the presence of thigh pain ranged 1 to 9 with a mean of 4.3. Mean stem subsidence was 2.7 ± 0.9 mm (range, 0–4.7 mm). The distribution of femoral stem alignment was all in a neutral position in the postoperative 3 days and the final follow-up AP hip radiographs. There were no significant differences in stem subsidence and alignment between the patients with and without occult periprosthetic femoral fracture. All 13 cases of occult intra-operative periprosthetic femoral fractures were healed at the final follow-up (Fig 6) without any additional surgical intervention.

Sex *(P =* .*01)* and CFR at 7 cm below the tip of the lesser trochanter *(P =* .*01)* were significantly associated with the occurrence of an occult intra-operative periprosthetic femoral fracture (Tables 2 and 3). After stepwise logistic regression analysis, only the sex was significantly associated with an increased risk in predicting an occult intra-operative periprosthetic femoral fracture (odds ratio of male, 0.25 compared with the female; 95% CI, 0.08–0.85; *p =* .*02*) (Table 4).

**Table 2:** Characteristics of risk factors with continuous variable for intra-operative periprosthetic femoral fracture during THA, including occult fractures.

Variable	Fracture (N = 16)	Non-fracture (N = 93)	p Value
**Age**	67 (63–72)	68 (64–70)	0.93
**BMI**^**a**^	24.9 (23-9-27.6)	24.2 (22.7–26.2)	0.31
**Stem size**	12 (10–16)	13 (9–16)	0.28
**Radiographic analysis**			
**CFI**^**b**^	3.91(3.42–4.55)	3.71 (3.14–4.06)	0.30
**CCR**^**c**^	0.48 (0.43–0.59)	0.53 (0.47–0.58)	0.25
**CBR**^**d**^	0.44 (0.39–0.50)	0.45 (0.40–0.49)	0.80
**CFR at ↑2 cm**^**e**^	57% (53%-66%)	55% (50%-60%)	0.13
**CFR at ↓7 cm**^**f**^	93% (90%-96%)	89% (85%-92%)	0.01^g^

^a^Body mass index

^**b**^**C**anal flare index

^**c**^Canal-calcar ratio

^**d**^Canal bone ratio

^**e**^Canal fill ratio at 2 cm above the tip of the lesser trochanter

^**f**^Canal filling ratio at 7 cm below the tip of the lesser trochanter

^g^p < 0.05

**Table 3:** Characteristics of risk factors with categorical variable for intra-operative periprosthetic femoral fracture during THA, including occult fractures.

Categorical variable	The incidence of fracture (%)		p Value
**Sex**			0.01^**c**^
**Male**	9/89 (10.1%)		
**Female**	7/20 (35.0%)		
**Diagnosis**			0.50
**ONFH**^**a**^	11/77 (14.3%)		
**Dysplastic hip**	4/13 (30.8%)		
**Osteoarthritis**	1/9 (11.1%)		
**PTOA**^**b**^	0/5 (0.0%)		
**Neck fracture**	0/5 (0.0%)		

^a^Osteonecrosis of the femoral head

^**b**^Posttraumatic osteoarthritis

^**c**^p < 0.05

**Table 4:** Simple and multiple logistic regression analysis of risk factors for intra-operative periprosthetic fracture during THA, including occult fractures.

Variable	Simple logistic regression					
	Coefficient (B)	SE^a^	Wald statistic	Odds ratio	95% CI	p Value
**Age**	NA	0.04	0.00	1	0.93–1.08	0.99
**Female**				1		
**Male**	-1.56	0.59	-2.67	0.21	0.07–0.67	^c^0.01
**BMI**	0.06	0.08	0.78	1.07	0.91–1.26	0.43
**ONFH**				1		
**Dysplastic hip**	0.98	0.68	1.44	0.40	0.02–2.23	0.39
**Osteoarthritis**	-0.29	1.11	-0.26	0.75	0.04–4.71	0.80
**PTOA**	-15.77	1769.25	-0.01	0.00	NA^b^	0.99
**Neck fracture**	-15.77	1769.25	-0.01	0.00	NA^b^	0.99
**Stem size**	0.10	0.08	1.18	1.10	0.93–1.30	0.21
**CFI**	0.41	0.35	1.17	1.50	0.75–3.00	0.24
**CCR**	-3.80	3.37	-1.13	0.02	NA^b^	0.26
**CBR**	-1.71	4.00	-0.43	0.18	NA^b^	0.67
**CFR at ↑2 cm**	7.39	4.14	1.78	1624.37	0.67–8879.73	0.11
**CFR at ↓7 cm**	6.13	3.80	1.61	459.10	2.48–2629.52	0.07
	Multiple logistic regression					
**Male**	-1.37	0.60	-2.27	0.25	0.07–0.84	^c^0.02
**CFR at ↓7 cm**	5.42	4.34	1.25	225.66	0.06–1696.10	0.21

^a^Standard error

^b^Not available

^**c**^p < 0.05

## Discussion

Periprosthetic occult fractures of the femur, to our knowledge, have not been evaluated previously using CT. We were interested to determine how often such occult fractures occurred because these un-displaced fractures might be a source of unexplained thigh pain after THA and are not typically detected during surgery or are not well seen on postoperative radiographs. We found the incidence of occult intra-operative periprosthetic femoral fracture (11.5%) was unexpectedly high, and in 9/13 (69.2%) of occult fractures, fracture lines were started from the region below the tip of the lesser trochanter. The female sex was associated with an increased risk of occult periprosthetic femoral fracture.

The present study has several limitations. First, we did not have bone density data for each patient with a preponderance of elderly patients (mean age, 66.4 years). Osteoporosis might have resulted in a higher-than-average risk of fracture in this population because weak bone in elderly people are well- known as an important risk factor for fractures. Second, we used only one type of femoral stem, and this may have affected the incidence of occult fracture in the present study because stem design would have a great influence on the outcomes including fractures. Tus, this study cannot be generalized to primary THA. Third, due to the small sample size of this study, especially a very small number of events, which makes it underpowered to address the determination of risk factors associated with occult fractures In cementless femoral stems, primary mechanical stability is achieved by means of press-fit which requires the bone to generate excessive periprosthetic strain. [27] A biomechanical study [28] showed that a conservative reaming procedure is beneficial to ensure sufficient primary mechanical stability without risking fracture. We performed a conservative reaming procedure, but the prevalence of occult intra-operative periprosthetic femoral fracture (11.5%) was relatively high. Our finding, combined with the findings of other reports in the acetabulum, [9, 10] suggests that the occurrence of occult periprosthetic fractures during press-fit should no longer be considered a rare event. We also found that in 9/13 (69.2%) of occult fractures, fracture lines were started from the region below the tip of the lesser trochanter. Because the region below the tip of the lesser trochanter which is covered by dense soft tissue is rarely exposure in cases of primary THAs, it can be difficult to detect an occult fracture during surgery and on postoperative plain radiographs.

With contemporary advances in CT technology and metal artifact reduction software, CT is preferable as the first line imaging modality for the investigation of post-operative THA patients when periprosthetic fractures are suspected. [29, 30] However, The routine use of CT scans in the detection of occult femoral fractures after THA may not be practical [10] or seems to be unnecessary due to the lack of clinical relevance. [11] There are some problems of gain visibility to detect occult fractures in primary THA during surgery or routine postoperative radiographs. In cases of displaced fractures, they would make a sudden change in resistance during insertion of the stem or unexplained instability during the stability check, which is highly suggestive of a femoral fracture or be easily visible on routine postoperative radiographs (Fig 3d). However, in cases of occult femoral fractures, due to the region below the tip of the lesser trochanter is covered by dense soft tissue and is rarely exposed during primary THA and fracture lines are involved only in the unilateral cortex without displacement, there is the problem of gain visibility to detect occult fractures during surgery or routine postoperative radiographs. In our study, the incidence of thigh pain was 15.0%, which was comparable with those of previous studies with a similar stem design. [31, 32] However, there was a significant difference in the occurrence of thigh pain between occult fracture group and non-occult fracture group *(P <* .*05)* and the median time of pain onset was different between the occult fracture group (median time; postoperative 7 days) and the non-occult fracture group (median time; postoperative 3 months). We found that out of the five hips that had detected femoral fractures around the lesser trochanter intraoperatively, four hips (80%) showed concurrent occult femoral fractures on different levels in the CT images. Based on our findings, we recommend a high index of suspicion and early CT referral for patients presenting with unexplained early postoperative thigh pain after cementless THA or intra-operative periprosthetic femoral fractures around the lesser trochanter were recognized during surgery.

We observed that the female sex was associated with an increased risk of occult intra-operative periprosthetic femoral fracture, but a higher CFR at 7 cm below the tip of the lesser trochanter shared some degree of interdependence (Table 4). Several previous studies [26, 33–35] suggested that the relationship between proximal femoral morphology and stem design may influence the outcome of the THA. Cooper et al. [33] mentioned that patients with a smaller CFI tended to have a greater degree of CFR in the mid and distal thirds. In contrast, Ishii et al. [26] observed the problem with the proximal-distal mismatch in patients with a greater CFI who had a greater degree of CFR in the mid and distal thirds due to the smaller size of the femoral canal. Considering our results and those of Cooper et al. [36] and Ishii et al., [26] surgeons should take proximal femoral bone morphology into consideration especially in female patients with stovepipe or champagne-flute morphology of the proximal femur to prevent occult intra-operative periprosthetic femoral fracture.

We found that there is no significant influence of occult fracture on stem subsidence and alignment at a minimum of 12 months follow-up. We also performed follow-up CT scans for some patients, which showed complete fracture healing (Fig 3). This finding is similar to that of the previous study. [9] Although the occult fractures did not affect the survival of the implant in our results, it is possible that such fractures might indicate a risk of early failure [36] if a secure fixation of the stem has not been confirmed intraoperatively.

## Conclusions

The authors suggest that occult intra-operative periprosthetic femoral fractures occur frequently during a long, trapezoidal, double-tapered cementless femoral stem (Corail^®^) fixation in primary THA, and that CT scans are helpful to detect them, and that these fractures may not adversely affect the survival of the implant if a rigid fixation of the implants has been achieved.

## Supporting information

S1 Dataset Minimal data set. (XLSX)
